# Perceptions of acceptance and reluctance to COVID-19 vaccination in Peru

**DOI:** 10.17843/rpmesp.2022.393.11337

**Published:** 2022-09-30

**Authors:** Janeth Tenorio-Mucha, Jill Portocarrero, Patricia Busta-Flores, M. Amalia Pesantes, María Lazo-Porras

**Affiliations:** 1 CONEVID, Knowledge and Evidence Unit, School of Medicine “Alberto Hurtado”, Universidad Peruana Cayetano Heredia, Lima, Peru. Universidad Peruana Cayetano Heredia CONEVID, Knowledge and Evidence Unit School of Medicine “Alberto Hurtado” Universidad Peruana Cayetano Heredia Lima Peru; 2 CRONICAS Center of Excellence in Chronic Diseases, Universidad Peruana Cayetano Heredia, Lima, Peru Universidad Peruana Cayetano Heredia CRONICAS Center of Excellence in Chronic Diseases Universidad Peruana Cayetano Heredia Lima Peru; 3 Dickinson College, Pennsylvania, United States of America. Dickinson College Dickinson College Pennsylvania USA; 4 Division of Tropical and Humanitarian Medicine, Geneva University Hospitals & University of Geneva, Geneva, Switzerland. University of Geneva Division of Tropical and Humanitarian Medicine Geneva University Hospitals & University of Geneva Geneva Switzerland

**Keywords:** COVID-19, COVID-19 vaccines, Vaccination Refusal, Health Belief Model

## Abstract

**Objectives.:**

To explore factors that influence the acceptance or reluctance to COVID-19 vaccination using qualitative methods.

**Materials and methods.:**

Descriptive qualitative study conducted between April and June 2021. A semi-structured interview guide was used to explore the perceptions of participants from different regions of Peru regarding COVID-19 vaccination. The Health Belief Model was used as theoretical framework and its dimensions are: susceptibility, severity, benefits, barriers, and cues to action.

**Results.:**

We interviewed 30 people, mostly were women. For the participants, the efficacy of vaccines is related to the country of origin of the vaccines; in addition, they consider that it is important to know the long-term effects on health after vaccination. The information received by governmental and health authorities can be a decisive factor for vaccination. People with the intention of not being vaccinated feel that vaccination promotion strategies violate their human rights.

**Conclusions.:**

There is a group of people undecided or unsure about receiving COVID-19 vaccines who need to be encouraged according to their concerns and needs. Governmental and health authorities should work together to improve the confidence of the population and provide messages to clarify doubts about the efficacy and adverse reactions of vaccines.

## INTRODUCTION

Vaccination is the most effective strategy to protect the population from several diseases, including COVID-19 ^1^. However, people have some reasons to hesitate about vaccination, which change over time as well as depending on the country and the region ^2^ ^-^ ^5^. According to Lane ^6^, the reasons why people decide to be vaccinated or not are marked by convenience, trust, and complacency towards health authorities. 

The first vaccine against COVID-19 was developed by Pfizer-BioNTech and offered 95% effectiveness against the disease ^7^; the United Kingdom was the first country to approve its use in early December 2020 ^8^. Vaccines arrived to Latin America on late December 2020. The first countries in the region to receive it were Mexico, Chile and Costa Rica ^9^. They arrived to Peru on February 8, 2021 and the first beneficiaries were front-line healthcare workers ^10^. One month later, on March 8, vaccination of the elderly began ^11^.

When vaccines began to be produced, middle- and low-income countries focused their efforts on acquiring them. It is in this context that we decided to conduct research that would allow us to understand the attitudes of the Peruvian population towards vaccination and to know their doubts or certainties regarding this issue. It is known that a particular challenge for COVID-19 vaccination is the excess of misinformation, which causes confusion, anxiety and fear about the origin and behavior of the disease, as well as uncertainty about the long-term effects of the vaccine ^12^ ^,^ ^13^. It is essential to understand the reasons why a given population is hesitant about vaccination, as this will help to identify strategies to promote acceptance. 

Peru has not only been one of the countries most affected by COVID-19 mortality ^14^, but also experienced a state of health emergency decreed on March 15, 2020 ^15^, which led to the initiation of a strict and long quarantine that lasted more than 100 days, until June 26, 2020 ^16^. The dissemination of a series of unproven treatments, even supported by healthcare professionals, contributed to mistrust among the population regarding the vaccine. On top of this, there was the political instability of a country that had three different presidents during a pandemic year and a scandal involving the secret vaccination of public officials outside a clinical trial ^17^.

By August 2021, most of the studies on the acceptability of vaccination against COVID-19 had been conducted in the United States and European countries, with some data reported in Bolivia, Ecuador, Brazil and Mexico ^13^. Although there are different studies to identify the factors associated with the acceptance of the COVID-19 vaccine, both in healthcare professionals and in the general population, very few have used qualitative methodology, especially in the Latin American population.

A better understanding of acceptance or reluctance to vaccination will allow the design of strategies or interventions based on community beliefs. Therefore, the aim of this study was to explore the factors that predispose to the acceptance or reluctance to COVID-19 vaccination in Peru.

**KEY MESSAGES**

Motivation for the study: a better understanding of reasons for acceptance or reluctance to COVID-19 vaccines will allow the design of strategies based on community beliefs, in order to increase acceptance and adherence to vaccination.

Main findings: vaccine reluctance is not only based on distrust of the vaccine, but also on the actions of the government, pharmaceutical companies and, above all, the media.

Implications: there are opportunities for intervention to stimulate vaccination, among them, the promotion of reliable information on efficacy and adverse events in the short and long term.

## MATERIALS AND METHODS

### Theoretical framework

**Figure 1:**
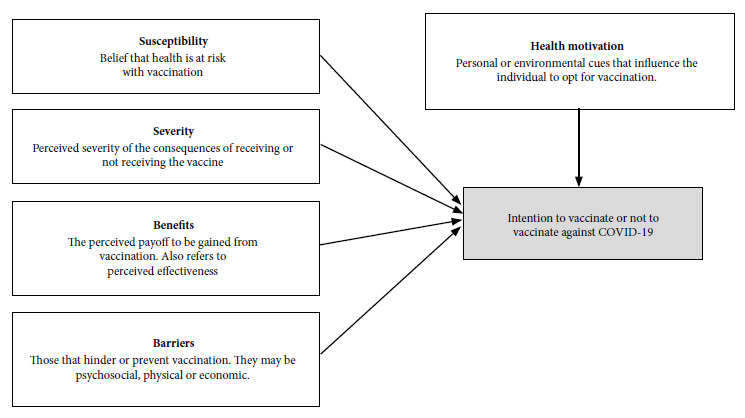
Model of health beliefs regarding the acceptance of vaccines against COVID-19.

We used the health belief model (HBM) as the theoretical framework of this research, which is based on the theory that individual beliefs and the perception of environmental conditions determine health-related behaviors ^18^. It comprises five constructs: perceived susceptibility; perceived severity; perceived benefits; perceived barriers; and health motivation (Figure 1). The HBM has been used to explore factors associated with vaccine acceptance for H1N1 ^19^ and, more recently, for the COVID-19 vaccine ^5^ ^,^ ^20^ ^,^ ^21^.

### Study design and context

Qualitative research using in-depth interviews. Qualitative research allows us to explore and understand the meanings that individuals attribute to a particular problem ^22^. This study seeks to understand the problem of acceptance and reluctance to vaccinate against COVID-19 from the particular perceptions of the Peruvian population. The in-depth interview allows the subjects to express their experiences and opinions in their own words ^23^.

The data for this study were collected when Peru was going through the second wave of the pandemic, with a high rate of positive cases. A total of 1.5 million doses of vaccine had been administered, but with uneven progress by age group and region, with Loreto, Puno, Ucayali, Madre de Dios and Amazonas being the most neglected regions ^24^.

### Participant selection

Participants were selected by purposive sampling. We selected individuals who were 18 years of age or older who did not receive any COVID-19 vaccine. Recruitment was conducted in two ways, first randomly selected from those who provided their data in an online survey conducted between March 9 and April 15, 2021. This survey aimed to conduct an initial quantitative exploration of the inclination to accept or refuse vaccines, and actions that might motivate vaccination. The survey was completed by 617 participants, most of whom were male (60.3%), lived in Lima (65.3%) and had higher education (89.3%). Of the total number of participants, 94.7% reported the inclination to be vaccinated. An invitation to participate in the in-depth interviews was included at the end of the survey and those interested provided their data. Two lists were then filtered, one with those with intention to be vaccinated and the other with those with no intention to be vaccinated. A recruiter from the research team reissued the invitation and those who gave their consent participated in the interviews.

We then we used snowball sampling due to the difficulties in reaching the number of participants for the group of those with no intention to be vaccinated; a participant extended the invitation to a private WhatsApp group of 30 to 50 members between men and women, who shared the same perceptions. In the group, the contact details of a study recruiter were provided, and those who were interested in participating contacted the recruiter who provided more detailed information about the study. Finally, those who gave their consent participated in the interviews. 

Recruitment was carried out by two health professionals who were also part of the research team. We invited 62 people through telephone calls or text messages, of whom 30 accepted and gave their consent. The main reasons for not participating were lack of time, refusal to provide personal data and rejection of the virtual format. Therefore, 15 participants who were inclined to accept the COVID-19 vaccine were recruited and 15 who were not.

### Procedures

### Elaboration of the interview guide

We followed a hybrid approach for the elaboration of the interview guide; first, deductively, questions were elaborated within the constructs of the HBM, and then, inductively, topics that emerged from the online survey were added. The initial development of the guide was carried out by a junior qualitative researcher, then the quantity and quality of the questions was discussed with the research team, and finally, a senior qualitative researcher reviewed the relevance of the guide. The questions explored knowledge and experience with COVID-19, reliability, and willingness to vaccinate, reasons for accepting or refusing vaccines, and actions that may encourage vaccination.

### Implementation of the interviews

All participants received a copy of the informed consent form in PDF by email or WhatsApp, and provided verbal consent (which was recorded) prior to the interview. The interviews were conducted via telephone or video call, according to the participant’s preference. We chose to collect information in this way in order to comply with the norms of social distancing and to reduce the risk of COVID-19 contagion among the interviewers and participants. This same strategy was used in similar studies carried out in other countries ^25^ ^-^ ^27^ and proved to be cost-effective, contributing to broaden the scope of the target population and creating a comfortable atmosphere between both parties (interviewer and interviewee) ^28^ ^,^ ^29^. The interviews were conducted by two researchers with previous experience in the use of qualitative techniques, and interviews were assigned according to time availability. Interviews were one-on-one, interviewer and interviewee, and lasted 40 min on average. The audio of the interview was recorded only with the participant’s consent. No participant opted to withdraw once the interview had begun, but they were aware that they could do so.

### Data analysis

The audios of the interviews were transcribed verbatim by personnel who did not participate in the elaboration of the guide or the data collection, and were reviewed by the research team. We used a thematic approach for the analysis, which allows to compare and contrast narratives between interviewees from the same group or between both groups. The reading of the transcripts allowed the identification of pre-established codes according to the HBM constructs. In addition, emergent codes that appeared in the transcripts were added. The code tree is attached as supplementary material. Coding was carried out by a single person, who developed the initial interview guide. The findings were discussed with the entire research team and reviewed by a senior researcher, but were not shared with the participants. Atlas Ti 7.5 software was used for the analysis. Findings are reported in narrative form and were accompanied by citations, organized according to the constructs of the CSM.

### Ethical and quality aspects

The study protocol, informed consent and instruments were approved by the Institutional Research Ethics Committee (CIEI) of the Universidad Peruana Cayetano Heredia with institutional code 204671. The information reported in this article complies with the guidelines of the Consolidated Criteria for Reporting Qualitative Research (COREQ) ^30^.

## RESULTS

Of the 30 participants, 21 were women; the average age was 33 years (minimum age 24 and maximum age 57). Most participants lived in Lima (n=21), the rest lived in Cusco, Ayacucho, Arequipa, and Ica.

### Perceived Susceptibility

Participants associated vaccine efficacy with the country of origin, regardless of their intention to vaccinate. There is greater perceived trust in vaccines from the United States and the United Kingdom, on the other hand vaccines from China and India are considered less trustworthy.

The participants mentioned that, from what was shown by the media, they knew about the immediate effects of vaccination, such as headache and fever, but no information was shown regarding long-term effects. This caused concern, because in their perception, vaccines could contribute to the development of diseases. Participants also reported concern regarding the effects of the vaccine on pregnant women, such as the reactions that the vaccine may produce in the development of the fetus or in the health of the pregnant woman.

*We have not been told what may happen in a few years to vaccinated people. It is not known whether it can cause any health problems, not now, but in five years or more. Nor do we know if, for example, what damage it may cause in pregnant women, in those children. Woman, intention of not applying the vaccine.*

### Perceived severity

Those who intended to be vaccinated mentioned that, if they were not vaccinated, COVID-19 infection could lead to very serious health problems and even death. Among these complications, they highlighted the presence of sequelae, and the impact on pre-existing health conditions such as asthma or diabetes.

### Perceived severity

Most of the participants who were inclined not to receive the vaccine believed that the COVID-19 virus was the same as other respiratory diseases and that the number of deaths from this virus was exaggerated. 

This same group considered that the measures taken by the Peruvian government for containment and prevention were exaggerated and violated their right to freedom. They also mentioned that the use of masks was not considered a solution, but rather a problem, because in the long run it could cause other diseases.

*(COVID-19) is no more different than any strong flu out there. The year before last 6 million people died in the world from pneumonia and nobody said anything. Now all those deaths are gone and they are being blamed on COVID-19. Man, intention not to take the vaccine.*

### Perceived benefits

Most of the participants who intended to be vaccinated were motivated mainly by individual and collective protection. Among other benefits, they mentioned the low probability of dying from the infection or suffering serious sequelae. They also recognized that vaccination would allow them to resume work and academic activities, in addition to reactivating activities that were interrupted by the state of emergency, such as receiving health care.

*I think the main benefit is that you do not develop the disease to a severe level. It is not going to take you to the ICU. They have all shown that, at least, it prevents you from developing the severe form, I think that is already a good indicator to take the vaccine that is available. Woman, intention to take the vaccine.*

On the other hand, although most of the participants who intended not to receive the vaccine did not perceive benefits for the individual, they did mention benefits at the macro level, such as the possibility of boosting economic or other activities that were affected by the long confinement experienced in the country. According to this group of participants, the government issued messages about the benefits of vaccination with the intention of boosting large-scale economic activities.

### Perceived barriers

On the one hand, most of the participants with the intention to be vaccinated mentioned that an important barrier to be considered, and that could affect the immunization process, is the lack of access to scientific information on vaccines, and that this would increase doubts and fears in the national population. They also mentioned that the shared information should be verified as it relates to beliefs about the COVID-19 virus and vaccine development.

*It is not fear, it is a real doubt that we should all be aware of. There is no clear information on how this virus started, some say by animals, people, or governments. We have no clear information on this, it is unlikely that we intend to trust vaccines then. Man, intention not to get the vaccine.*

On the other hand, the people with no intention of being vaccinated mentioned that their decision could be undermined if the government imposed mandatory vaccination, since this was related to their autonomy. In addition, they considered that the Peruvian State made the population believe that the only “cure” for the COVID-19 virus was vaccination, therefore, people who did not intend to be vaccinated would be considered as not interested in the health of others. Although part of this group acknowledged that they would be the ones most at risk of infection and that they respected the decision of those who get vaccinated, but reaffirmed their position of not getting vaccinated.

### Health motivation

A common theme among both groups of participants was the use of the media to inform themselves about vaccines. Some believed that the broadcast media (television and radio) sought to generate alarm in the population by showing the number of deaths and stories of economic loss. For participants who did not intend to receive the vaccine, they felt that these same traditional media outlets provided biased information in favor of the vaccine.

*The Peruvian press is subject to what money does. I have seen in the news that there is too much sad information. They boast too much about something that is small. They really give you the news to alarm you, but they do not give you the news so that you proceed and take care of yourself. Man, intention to get the vaccine.*

Finally, we found that the social environment was a motivation for participants. On the one hand, the participants who had the intention of being vaccinated mentioned that the decision was totally personal, but that witnessing the vaccination of people close to them and, in addition, observing the reports on the efficacy, produced in them greater acceptance to vaccination. On the other hand, participants who did not intend to be vaccinated mentioned that they did not feel isolated in this perception. Although they considered that the media sought to “stigmatize” them for this decision, they indicated that their support networks showed them that waiting or not intending to get vaccinated were the right decisions.

## DISCUSSION

This study found that, although there was dissemination about the immediate adverse events of vaccination, there is a lack of knowledge about the long-term effects. The fear is shared by the general population and has also been reported for other vaccines that have been developed in recent years ^31^ ^,^ ^32^. But it is even greater for the COVID-19 vaccine because of the rapid vaccine manufacturing process and the short time of sanitary clearance conferred. A review on the origin and composition of COVID-19 vaccines and their correlation with other existing vaccines reveals that the expected immune and autoimmune events are very rare and nonlethal ^33^. Information on the long-term effects of vaccines will be generated as a result of the reports of events supposedly attributed to vaccination that all countries, including Peru, are providing. The dissemination of medical evidence, using simple terms, on the expected long-term events and on the possibility of reporting adverse events could help to increase confidence in vaccines against COVID-19. It is also necessary to emphasize research on long-term effects in vulnerable populations such as pregnant women, fetuses, children, and the elderly. 

The reasons reported by people with no intention to be vaccinated against COVID-19 are not only based on distrust of the vaccine, but also on distrust of the actions of the government, pharmaceutical companies and, above all, the traditional media, such as the news broadcasts. These results are similar to those described in qualitative and quantitative studies carried out in other countries ^25^ ^,^ ^34^, where the information received by health agencies about future risks is a main predictor of vaccine acceptance or resistance. Therefore, health authorities should take an active role and develop rapid responses to debunk misinformation and myths, with messages tailored to different population groups, in addition to addressing population concerns such as long-term effects or during pregnancy. The information provided should be consistent so that it is not overshadowed by conflicts of interest that could undermine the vaccination strategy ^35^.

An interesting finding of this study is the reported distrust of traditional media such as radio and television. People with the intention of not being vaccinated perceived that the media handled information about COVID-19 in a way that was misleading and biased towards vaccination. To address this problem, we recommend a synergistic work between the media, the scientific community, and religious institutions to build and disseminate relevant communication messages. To this end, we can learn from previous experiences in vaccination, such as the use of culturally appropriate videos that tell stories that address specific beliefs and behaviors about the susceptibility and severity of the disease, as well as the effectiveness of prevention, which identify participants and improve vaccination coverage ^36^ ^,^ ^37^.

Identifying the factors related to acceptance or reluctance is key, especially to work on those factors that are modifiable (e.g. knowledge about the possibility of reinfection) and thus increase the acceptance of vaccination. But it is also relevant to identify those factors that are not modifiable (e.g. beliefs), in order to know which groups are the most reluctant and to be able to work on specific strategies with them and for them.

This study is relevant for the Peruvian context, but it can also be useful for the Latin American and global context. Our results can be used to intervene and design strategies focused on the fears and doubts of the population. Likewise, the use of qualitative methodology can be replicated in other contexts to identify specific fears or doubts related to COVID-19 vaccination, as well as reluctance to use vaccines in general.

The qualitative design of this study allowed an in-depth exploration of the reasons for acceptance and reluctance to vaccinate against COVID-19. However, there are some limitations that should be mentioned. First, it is highly likely that the snowball recruited group shared the same perceptions and that our findings on intention not to be vaccinated are targeted to that particular group. Second, remote data collection may have influenced trust between interviewer and interviewee, nonetheless, it was considered to be the safest method because of the ongoing pandemic. Third, coding was conducted by a single person and only the findings were discussed with the research group, but the findings were not shared with the interviewees, nor was a further step taken to evidence the value of the findings. Finally, we consider the lack of reliability due to the lack of triangulation in the methodology as another limitation.

In conclusion, the main fear of COVID-19 vaccines is related to long-term adverse events. There are reasons for vaccination mistrust that are not directly related to vaccines, but derive from mistrust of authorities, health institutions and the media. To increase the willingness to vaccinate, messages with clear, reliable, and culturally appropriate information could be provided and should be worked together with institutions directly related to public health promotion.
