# Supplementary material for: Perceptions of acceptance and reluctance to COVID-19 vaccination in Peru
Source: Rev Peru Med Exp Salud Publica. 2022 Sep 30;39(3):274–80. doi: 10.17843/rpmesp.2022.393.11337 (PMC11397679; doi:10.17843/rpmesp.2022.393.11337)
Supplement: Translation

# Translation: Percepciones de aceptabilidad y reticencia a las vacunas contra la COVID-19 en el Perú

**Authors:** Janeth Tenorio-Mucha, Jill Portocarrero, Patricia Busta-Flores, M. Amalia Pesantes, María Lazo-Porras

**Affiliations:** 1 CONEVID, Unidad de Conocimiento y Evidencia, Facultad de Medicina «Alberto Hurtado», Universidad Peruana Cayetano Heredia, Lima, Perú; 2 CRONICAS Centro de Excelencia en Enfermedades Crónicas, Universidad Peruana Cayetano Heredia, Lima, Perú; 3 Dickinson College, Pennsylvania, United States of America.; 4 Division of Tropical and Humanitarian Medicine, Geneva University Hospitals & University of Geneva, Geneva, Switzerland.

**Keywords:** COVID-19, Vacunas contra la COVID-19, Negativa a la Vacunación, Modelo de Creencias sobre Salud

## Abstract

**Objetivos.:**

Explorar los factores que predisponen la aceptabilidad o reticencia a la vacunación contra la COVID-19 utilizando métodos cualitativos.

**Materiales y métodos.:**

Estudio cualitativo descriptivo realizado entre abril y junio del 2021. Se utilizó una guía de entrevista semiestructurada que exploró las percepciones de los participantes de diferentes regiones del Perú con relación a la aplicación de la vacuna para la COVID-19, tomando como marco teórico el modelo de creencias en salud, cuyas dimensiones son: susceptibilidad, severidad, beneficios, barreras y estímulos asociados para ejecutar la acción.

**Resultados.:**

Se entrevistó a treinta personas, en su mayoría mujeres. Para los participantes la eficacia de las vacunas se relaciona con el país de procedencia, además, consideran que es importante conocer los efectos a largo plazo en la salud luego de la aplicación de la vacuna. La información recibida por las autoridades gubernamentales y sanitarias puede ser un factor decisivo para la aplicación de la vacuna. Las personas con la intención de no vacunarse sienten que las estrategias de promoción de la vacunación vulneran sus derechos humanos.

**Conclusiones.:**

Existe un grupo de personas indecisas o inseguras de recibir las vacunas contra la COVID-19 en quienes es necesario fomentar estímulos de acuerdo con sus preocupaciones y necesidades. Las autoridades gubernamentales y sanitarias deben trabajar conjuntamente para mejorar la confianza de la población y brindar mensajes que despejen las principales dudas sobre la eficacia y las reacciones adversas de las vacunas.

## INTRODUCCIÓN

La vacunación es la estrategia más efectiva para proteger a la población de muchas enfermedades, entre ellas de la COVID-19^ (^ ^1^. Pero, las personas tienen diversas razones que les ocasionan dudas frente a la vacunación y estas varían dependiendo de la región, del país y, además, cambian con el tiempo ^2^ ^-^ ^5^. Según Lane ^6^, las razones por las que las personas deciden vacunarse o no están marcadas por la conveniencia, la confianza y la complacencia frente a las autoridades en salud. 

La primera vacuna contra la COVID-19 fue elaborada por Pfizer-BioNTech, que ofrecía un 95% de efectividad contra la enfermedad ^7^ y fue Reino Unido el primer país en aprobar su uso a inicios de diciembre del 2020 ^8^. En Latinoamérica, la llegada de las vacunas comenzó a finales de diciembre del 2020. Los primeros países de la región en recibirla fueron México, Chile y Costa Rica ^9^. Al Perú llegaron el 8 de febrero del 2021 y los primeros beneficiarios fueron los trabajadores de salud de la primera línea ^10^. Un mes después, el 8 de marzo, se inició la vacunación en los adultos mayores ^11^.

Cuando las vacunas empezaron a ser producidas, los países de ingresos medios y bajos enfocaron sus esfuerzos en procurar que esas vacunas llegaran a sus países. Es en este contexto en el que decidimos realizar una investigación que nos permitiera comprender las actitudes de la población peruana hacia la vacunación y conocer sus dudas o certezas con relación a este tema. Se sabe que un reto particular de la vacunación para la COVID-19 es la sobrecarga de desinformación, lo cual causa confusión, ansiedad y miedo sobre el origen y comportamiento de la enfermedad, así como incertidumbre de los efectos de la vacuna a largo plazo ^12^ ^,^ ^13^. Es esencial comprender las razones por las que una determinada población tiene dudas frente a la vacuna, pues ello permitirá saber cuáles son las estrategias para promover la aceptabilidad. 

Perú no solo ha sido uno de los países más afectados por la mortalidad de la COVID-19 ^14^, sino que también experimentó un estado de emergencia sanitaria que se decretó el 15 de marzo de 2020^15^, y que conllevó al inicio de una estricta y larga cuarentena que se prolongó por más de 100 días, hasta el 26 de junio de 2020 ^16^. La difusión de una serie de tratamientos no comprobados, respaldados incluso por profesionales de la salud, contribuyeron a una sensación de desconfianza de la población con relación a la vacuna. A ello se le suma la inestabilidad política de un país que tuvo tres presidentes distintos durante un año de pandemia y un escándalo que involucró la vacunación secreta de funcionarios públicos por fuera de un ensayo clínico ^17^.

Para agosto del 2021, la mayoría de estudios reportados sobre la aceptabilidad de la vacunación contra la COVID-19 se habían realizado en Estados Unidos y países europeos, con algunos datos reportados en Bolivia, Ecuador, Brasil y México ^13^. Si bien existen diferentes estudios para identificar los factores asociados con la aceptación de la vacuna contra la COVID-19, tanto en profesionales de salud, como en la población en general, son muy pocos los que han utilizado metodología cualitativa, especialmente en la población latinoamericana. 

Un mejor entendimiento de la aceptabilidad o reticencia a la vacunación permitirá el diseño de estrategias o intervenciones basadas en las creencias de la comunidad. Por lo tanto, el objetivo de este estudio fue explorar los factores que predisponen la aceptabilidad o reticencia a las vacunas contra la COVID-19 en el Perú.

**MENSAJE CLAVE**

Motivación para realizar el estudio: un mejor entendimiento de razones para la aceptabilidad o reticencia a las vacunas contra la COVID-19 permitirá el diseño de estrategias basadas en las creencias de la comunidad para incrementar la aceptación y adherencia a la vacunación.

Principales hallazgos: la reticencia hacia la vacuna no solo está centrada en la desconfianza hacia la vacuna, sino que también en las acciones del gobierno, las empresas farmacéuticas y, sobre todo, en los medios de comunicación.

Implicancias: existen oportunidades de intervención para estimular la vacunación, entre ellas, la promoción de información confiable sobre la eficacia y eventos adversos a corto y largo plazo.

## MATERIALES Y MÉTODOS

### Marco teórico

El marco teórico de esta investigación corresponde al modelo de creencias en salud (MCS), este se basa en la teoría de que las creencias individuales y la percepción de las condiciones del entorno determinan las conductas relacionadas con la salud ^18^. Comprende cinco constructos: susceptibilidad percibida; severidad percibida; beneficios percibidos; barreras percibidas y estímulos para ejecutar la acción (Figura 1). El MCS ha sido usado para explorar los factores asociados con la aceptación de vacunas para la gripe H1N1 ^19^ y, más recientemente, para la vacuna contra la COVID-19 ^5^ ^,^ ^20^ ^,^ ^21^.

**Figura 1:** Modelo de creencias en salud en el marco de la aceptabilidad a las vacunas contra la COVID-19.

### Diseño y contexto del estudio

Investigación cualitativa utilizando entrevistas a profundidad. La investigación cualitativa permite explorar y entender los significados que los individuos atribuyen a un problema en particular ^22^. En este estudio se busca entender el problema de la aceptabilidad y la reticencia a la vacunación contra la COVID-19 desde las percepciones particulares de la población peruana. La entrevista a profundidad permite que los sujetos expresen con sus propias palabras sus experiencias y opiniones ^23^. 

Durante la obtención de los datos para este estudio, Perú atravesaba la segunda ola, con una alta tasa de casos positivos. Se habían aplicado 1,5 millones de dosis de vacunas, pero con un avance desigual por grupos etarios y por regiones, siendo Loreto, Puno, Ucayali, Madre de Dios y Amazonas, las más regiones más relegadas ^24^.

### Selección de participantes

Los participantes fueron seleccionados por muestreo intencional. Las personas elegibles debían ser mayores de 18 años y no haber recibido ninguna vacuna contra la COVID-19. El reclutamiento se realizó de dos formas, primero se eligió aleatoriamente entre aquellos que proporcionaron sus datos en una encuesta en línea realizada entre el 9 de marzo y el 15 de abril del 2021. Esta encuesta tuvo como objetivo realizar una exploración cuantitativa inicial sobre la inclinación para aceptar o rechazar las vacunas, y las acciones que podrían motivar la vacunación. La pesquisa fue completada por 617 participantes, la mayoría de sexo masculino (60,3%), con residencia en Lima (65,3%) y con educación superior (89,3%). El 94,7% indicó tener inclinación a vacunarse. Al final de la encuesta se dejó una invitación para participar de las entrevistas a profundidad y los interesados dejaron sus datos. Luego se filtraron dos listas, una con la intención de vacunarse y la otra con la intención de no vacunarse. Un reclutador del equipo de investigación volvió a realizar la invitación y quienes dieron su consentimiento, participaron de las entrevistas. 

Luego, debido a las dificultades para alcanzar el número de participantes del grupo con intención de no vacunarse, se utilizó un muestreo por bola de nieve, una participante extendió la invitación a un grupo privado de WhatsApp, de entre 30 a 50 miembros entre hombres y mujeres, que compartían las mismas percepciones. En el grupo se dejó los datos de contacto de un reclutador del estudio, quienes tuvieron interés de participar se comunicaron con el reclutador quien brindó información más detallada del estudio. Finalmente, quienes dieron su consentimiento, participaron de las entrevistas. 

El reclutamiento fue realizado por dos profesionales de salud que también eran parte del equipo de investigación. Mediante llamadas telefónicas o mensajes de texto, se invitó a 62 personas, de las cuales 30 aceptaron y dieron su consentimiento. Los principales motivos para no participar fueron la falta de tiempo, rechazo a brindar datos personales y rechazo al formato virtual. De modo equitativo, se reclutaron 15 participantes que se inclinaban por la aceptación de las vacunas contra la COVID-19 y 15 que no. 

### Procedimientos

### Elaboración de la guía de entrevista

La elaboración de la guía de entrevista siguió un enfoque hibrido; primero, deductivamente se elaboraron preguntas dentro de los constructos del MCS, y luego, inductivamente, se añadieron tópicos que emergieron de la encuesta en línea. La elaboración inicial de la guía fue realizada una investigadora cualitativa junior, luego se discutió la cantidad y calidad de las preguntas con el equipo de investigación y, finalmente, una investigadora cualitativa senior revisó la pertinencia de la guia. Las preguntas exploraron conocimientos y experiencias sobre la COVID-19, confiabilidad y disposición para vacunarse, razones para aceptar o rechazar las vacunas y las acciones que pueden fomentar la vacunación. 

### Aplicación de las entrevistas

Todos los participantes recibieron una copia del consentimiento informado del estudio en versión PDF por correo electrónico o WhatsApp, y brindaron su consentimiento verbal (que fue grabado) previo a la entrevista. Las entrevistas fueron realizadas vía telefónica o videollamada, según preferencia del participante. Se optó por recolectar información de esta manera, en respeto a las normas de distanciamiento social y para disminuir el riesgo de contagio de la COVID-19 en las entrevistadoras y los participantes. Esta misma estrategia fue utilizada en estudios con un objetivo similar al nuestro realizado en otros países ^25^ ^-^ ^27^ y que resultaron ser costo-efectivas, contribuyen a ampliar el alcance de la población objetivo y propician un ambiente de comodidad entre ambas parte (entrevistador y entrevistado) ^28^ ^,^ ^29^. Las entrevistas fueron realizadas por dos investigadoras con experiencia previa en el uso de técnicas cualitativas, la asignación de entrevistas se realizó por disponibilidad de horario. Las entrevistas fueron uno-a-uno, entrevistador y entrevistado, y duraron 40 min en promedio. Con el consentimiento del participante se grabó el audio de la entrevista en grabadoras de voz. Ningún participante optó por retirarse una vez iniciada la entrevista, pero tenían conocimiento que podían hacerlo si lo consideraban pertinente. 

### Análisis de datos

Los audios de las entrevistas fueron transcritos textualmente por personal que no participó de la elaboración de la guía ni de la recolección de datos, y fueron revisadas por el equipo de investigación. Para el análisis se utilizó un enfoque temático, el cual permite comparar y contrastar las narraciones entre los entrevistados de un mismo grupo o entre ambos. La lectura de las transcripciones permitió la identificación de los códigos preestablecidos según los constructos del MCS. Además, se agregaron códigos emergentes que aparecieron en las transcripciones. El árbol de códigos se adjunta como material suplementario. La codificación estuvo a cargo de una sola persona, la cual elaboró la guía inicial de entrevista. Los hallazgos se discutieron con todo el equipo de investigación y fueron revisados por una investigadora senior, pero no se compartieron con los participantes. Para el análisis se utilizó el *software* Atlas Ti 7.5. Los hallazgos se reportan de manera narrativa y se acompañaron de citas, organizadas según los constructos del MCS. 

### Aspectos éticos y de calidad

El protocolo del estudio, consentimiento informado e instrumentos fueron aprobados por el Comité Institucional de Ética en Investigación (CIEI) de la Universidad Peruana Cayetano Heredia con código institucional 204671. Lo reportado en este artículo se ajusta a las directrices del Consolidated Criteria for Reporting Qualitative Research (COREQ) ^30^. 

## RESULTADOS

De los 30 participantes, 21 eran mujeres; la edad promedio fue de 33 años (edad mínima 24 y máxima 57). La mayoría de los participantes indicaron su residencia en Lima (n=21), mientras que los demás indicaron su residencia en Cusco, Ayacucho, Arequipa e Ica.

### Susceptibilidad percibida

Los participantes asociaron la eficacia de las vacunas con el país de procedencia, independiente de su intención sobre la vacunación. Existe mayor percepción de confianza de las vacunas procedentes de Estados Unidos y el Reino Unido, y se consideran como menos confiables a las vacunas procedentes de China e India. 

Los participantes mencionaron que, por lo mostrado en los medios de comunicación, se conocía sobre los efectos inmediatos a la aplicación, como dolor de cabeza y fiebre, pero no se mostraba información sobre los efectos a largo plazo. Esto generaba preocupación, pues en su percepción, podrían contribuir al desarrollo de enfermedades. Otra preocupación reportada fue sobre los efectos de la vacuna en mujeres gestantes, como las reacciones que la vacuna pueda producir en el desarrollo del feto o en la salud de la gestante. 

*No nos han dicho qué puede suceder dentro de unos años en las personas vacunadas. No se sabe si te puede dar algún problema de salud, no ahora, sino en cinco años o más. Tampoco se sabe si, por ejemplo, qué daños puede generar en mujeres embarazadas, en esos niños.* Mujer, intención de no aplicarse la vacuna.

### Severidad percibida

Entre aquellos con inclinación a vacunarse mencionaron que, de no estar vacunados, la infección por la COVID-19 podría generar problemas de salud muy serios e incluso la muerte. Entre estas complicaciones resaltan la presencia de secuelas, el impacto en condiciones de salud preexistentes como asma o diabetes. 

La mayoría de los participantes con inclinación a no recibir la vacuna creen que el virus de la COVID-19 es igual al de otras enfermedades respiratorias y que las cifras de fallecidos por este virus eran exageradas. Este mismo grupo consideró que las medidas tomadas por el gobierno peruano para la contención y prevención eran exageradas y vulneraban su derecho a la libertad. Asimismo, mencionaron que el uso de mascarilla no era considerado como una solución, sino más bien un problema, porque a la larga podría generar otras enfermedades.

*(La COVID-19) no es más diferente que cualquier gripe fuerte que exista. El anteaño pasado murieron 6 millones de personas en el mundo por pulmonía y nadie dijo nada. Ahora todos esos muertos han desaparecido y se los echan a cargo al COVID-19. *Hombre, intención de no aplicarse la vacuna.

### Beneficios percibidos

La mayoría de los participantes con intención a recibir la vacuna estaban motivados, principalmente, por la protección individual y colectiva. Entre otros beneficios, se mencionó la poca probabilidad de fallecer a causa de la infección o de sufrir secuelas graves. También reconocieron que la vacunación permitiría retomar actividades presenciales laborales y académicas, además de reactivar actividades que se interrumpieron por el estado de emergencia, como recibir atención en salud. 

*Creo que el principal beneficio es que no desarrollas la enfermedad a un nivel grave. No te va llevar a UCI. Todas han demostrado que, al menos, previene que uno no llegue a desarrollar la manera grave, creo que ya es un buen indicador para tomar la vacuna que esté a disposición. *Mujer, intención de aplicarse la vacuna.

Por otro lado, si bien la mayoría de los participantes con intención de no recibir la vacuna no percibían beneficios para la persona, sí mencionaron beneficios a nivel macro, como la posibilidad de repotenciar las actividades económicas u otras actividades que fueron afectadas por el largo confinamiento experimentado en el país. Según este grupo de participantes, los gobernantes emitían mensajes sobre los beneficios de la vacunación con la intención de impulsar actividades económicas de gran escala.

### Barreras percibidas

Por un lado, la mayoría de los participantes con intención de vacunarse mencionaron que una barrera importante a tomar en cuenta, y que podría afectar el proceso de inmunización, es el poco acceso a la información científica sobre las vacunas, y que esto incrementaría las dudas y temores en la población nacional. Asimismo, mencionaron que la información compartida debería ser verificada ya que eso guarda relación sobre las creencias en torno al virus de la COVID-19 y al desarrollo de vacunas. 

*No es temor, es una duda real que todos deberíamos conocer. No hay información clara de cómo se inició este virus, algunos dicen que por animales, personas o gobiernos. No tenemos información clara sobre esto, es improbable que pretendamos confiar en las vacunas entonces. *Hombre, intención de no aplicarse la vacuna.

Por otro lado, dentro del grupo con intención de no recibir las vacunas, mencionaron que su decisión podría verse vulnerada si el gobierno impone la obligatoriedad a la vacunación ya que esto iba relacionado con su autonomía. Además, consideraban que el Estado Peruano hizo creer a la población que la única «cura» para el virus de la COVID-19 era la vacunación, por lo tanto, las personas que no tuvieran la intención de vacunarse serían consideradas como no interesadas en la salud de los otros. Aunque una parte de este grupo reconoció que ellos serían los que tendrían mayor riesgo ante el contagio y que respetaban la decisión de quienes se vacunan, pero se reafirmaban en su posición de no vacunarse.

### Estímulos para ejecutar la acción

Un punto común entre ambos grupos de participantes fue el uso de medios de comunicación para informarse sobre las vacunas. Algunos creían que los medios de comunicación de señal abierta (televisión y radio) buscaban generar alarma en la población al mostrar la cifra de fallecidos e historias de pérdida económica. Para los participantes que no tenían la intención de recibir la vacuna, consideraban que estos mismos medios de comunicación tradicionales brindaban información parcializada a favor de la vacuna.

*La prensa peruana está sujeta a lo que el dinero hace. Yo he visto en los noticieros que existe demasiado información triste. Alardean demasiado una cosa que es pequeña. Verdaderamente dan la noticia como para alarmarte, pero no te dan una noticia para que tu procedas y te cuides.* Hombre, intención de aplicarse la vacuna.

Por último, el entorno social fue visto como un estímulo que considerar para los participantes. Por un lado, los participantes que tenían la intención de recibir la vacuna mencionaron que la decisión era totalmente personal, pero que presenciar la vacunación de personas cercanas y, además, observar los reportes sobre la eficacia, generaba en ellos mayor aceptabilidad a la aplicación. Por otro lado, dentro del grupo de personas que no tenían la intención de vacunarse mencionaron que no se sentían aislados al tener esa percepción. Si bien consideraban que los medios de comunicación buscaban «estigmatizarlos» por esa decisión, indicaron que sus redes de apoyo les demostraba que el esperar o no tener la intención de vacunarse eran las decisiones adecuadas.

## DISCUSIÓN

Este estudio encontró que, si bien hubo difusión sobre los eventos adversos inmediatos de la vacunación, hay desconocimiento de los efectos a largo plazo. Este temor es compartido por la población en general y también ha sido reportado para otras vacunas que se han desarrollado en los últimos años ^31^ ^,^ ^32^. Pero es aún mayor para la vacuna contra la COVID-19 debido al rápido proceso de manufactura de la vacuna y el poco tiempo de autorización sanitaria conferido. Una revisión sobre el origen y composición de las vacunas contra la COVID-19 y su correlación con otras vacunas existentes, revela que los eventos inmunes y autoinmunes esperados son muy raros y no son letales ^33^. La información sobre los efectos a largo plazo de las vacunas se irá generando como producto de los reportes de eventos supuestamente atribuidos a la vacunación que todos los países, incluido el Perú, están generando. La difusión de la evidencia médica, con un lenguaje sencillo, sobre los eventos esperados a largo plazo y sobre la posibilidad de reportar eventos adversos podría ayudar a incrementar la confianza en las vacunas contra la COVID-19. Es necesario, también, poner énfasis en investigaciones que generen información sobre efectos a largo plazo en poblaciones vulnerables como gestantes, fetos, niños y adultos mayores. 

Las razones reportadas por las personas con la inclinación de no vacunarse contra la COVID-19, no solo están centradas en la desconfianza a la vacuna, sino también en la desconfianza a las acciones del gobierno, las empresas farmacéuticas y, sobre todo, a los medios de comunicación tradicionales, como los noticieros. Estos resultados son similares a lo descrito en estudios cualitativos y cuantitativos realizados en otros países ^25^ ^,^ ^34^ donde la información recibida por los organismos sanitarios en torno a los riesgos a futuro es un predictor principal para la aceptación o resistencia a la vacuna. Es por eso que, las autoridades sanitarias deben tomar un rol activo y desarrollar respuestas rápidas para desmentir información falsa y mitos, con mensajes adaptados a diferentes grupos de la población, además, de abordar las preocupaciones de la población como, por ejemplo, efectos a largo plazo o durante el embarazo. La información que se brinde deber ser consistente para que no se vea opacada por conflictos de intereses que podrían perjudicar la estrategia de vacunación ^35^. 

Un hallazgo interesante de este estudio es la desconfianza reportada hacia los medios de comunicación tradicionales como la radio y la televisión. Las personas con intención de no vacunarse percibían que los medios manejaban información sobre la COVID-19 que era engañosa y que estaba parcializada hacia la vacunación. Para abordar este problema es recomendable un trabajo sinérgico entre los medios de comunicación, la comunidad científica y las instituciones religiosas, para construir y difundir mensajes comunicacionales pertinentes. Para ello, podemos aprender de experiencias previas en vacunación, como por ejemplo, el uso de videos culturalmente apropiados en los que se cuentan historias que abordan creencias y comportamientos específicos sobre la susceptibilidad y severidad de la enfermad, así como la eficacia de la prevención, los cuales identifican a los participantes y mejorar la cobertura de vacunación ^36^ ^,^ ^37^. 

Identificar los factores relacionados con la aceptabilidad o reticencia es clave, especialmente para trabajar en aquellos factores que son modificables (p. ej. conocimiento sobre la posibilidad de reinfectarse) y con ello lograr que la vacuna sea más aceptada. Pero también es relevante el identificar aquellos factores no modificables (p. ej. creencias), para así conocer qué grupos son aquellos con mayor reticencia y poder trabajar estrategias específicas con ellos y para ellos.

Este estudio es relevante para el contexto peruano, pero también puede ser de utilidad para el contexto Latinoamericano y global, en primer lugar porque la información encontrada puede ser utilizada para intervenir y diseñar estrategias enfocadas en los miedos y dudas de la población y, en segundo lugar, por la metodología utilizada; el uso de metodología cualitativa puede ser replicado por otros contextos para identificar miedos o dudas específicas relacionadas con la vacunación por la COVID-19, así como la reticencia al uso de las vacunas en general.

El diseño cualitativo de este estudio nos permitió una exploración profunda sobre los motivos para la aceptación y reticencia a la vacunación para la COVID-19. Sin embargo, existen algunas limitaciones que se deben mencionar. Primero, en el grupo reclutado por bola de nieve es altamente probable que compartan las mismas percepciones y que nuestros hallazgos sobre la intención de no vacunarse estén dirigidos a ese grupo en particular. Segundo, la recolección de datos de manera remota pudo haber influenciado en la confianza entre el entrevistador y entrevistado, pero se consideró como el método más seguro debido a la pandemia. Tercero, la codificación fue realizada por una sola persona y solo se discutieron los hallazgos con el grupo de investigación, pero no se compartieron los hallazgos con los entrevistados, ni se realizó otro paso posterior para evidenciar el valor de estos. Por último, se reconoce la limitación de falta de confiabilidad por la falta de triangulación en la metodología. 

En conclusión, el principal temor percibido hacia las vacunas contra la COVID-19 está relacionado con los eventos adversos a largo plazo. Existen razones para la desconfianza a las vacunas que no están directamente relacionadas con ellas, sino que derivan de la desconfianza de las autoridades, instituciones sanitarias y los medios de comunicación. Para incrementar la disposición hacia la vacunación se podrían brindar mensajes con información clara, confiable y culturalmente apropiada que debe ser trabajada en conjunto con instituciones directamente relacionadas con la promoción de salud pública.
